# Bibliometric analysis of lipophagy:2013 to 2023

**DOI:** 10.1016/j.heliyon.2024.e35299

**Published:** 2024-08-02

**Authors:** Lu Zhao, Mengmeng Pang, Zhenyue Fu, Huaqin Wu, Qingqiao Song

**Affiliations:** a Department of Cardiology, Guang'anmen Hospital, China Academy of Chinese Medical Sciences, 5 Beixiange, Xicheng District, Beijing, 100053, China; b Beijing University of Chinese Medicine, Beijing, China; c Department of General Medicine, Guang'anmen Hospital, China Academy of Chinese Medical Sciences, 5 Beixiange, Xicheng District, Beijing, 100053, China

**Keywords:** Lipophagy_1_, Bibliometric_2_, Lipid metabolism_3_, Fatty liver_4_, Atherosclerosis_5_, Cancer_6_

## Abstract

Lipophagy is defined as the autophagic degradation of lipid droplets. It is a selective autophagy process that can continuously circulate and redistribute metabolites to maintain the body's energy balance. Over the last ten years, there has been a significant increase in the amount of literature on lipophagy, making it more challenging to track the field's advancement using conventional techniques. The data from the lipophagy literature published in the last ten years was converted into visual representations with the use of bibliometric tools. An increasing number of countries and institutions are delving further into lipophagy research with the support of visualization technologies. The five main illnesses of cancer, atherosclerosis, fatty liver, hyperlipidemia, and neurodegenerative diseases have become study opportunities, as have the mechanisms of macroautophagy, microautophagy, and chaperone-mediated autophagy.

## Highlights

• This article uses bibliometrics to sort out the literature on lipophagy in the past decade.
• Investigate the history, frontiers, and most recent developments in lipophagy research by using visual knowledge networks and maps.
• It was found that various countries and institutions have increasingly conducted more and more in-depth research on lipophagy.
• The three forms of autophagy—macroautophagy, microautophagy, and chaperone-mediated autophagy—have demonstrated their potential therapeutic in cancer, atherosclerosis, fatty liver, hyperlipidemia, and neurodegenerative diseases.

## 1 Introduction

Lipid droplets (LDs) exist in adipocytes as an essential organelle for intracellular storage of lipids. These dynamic organelles participate in cell metabolism and control energy balance by expanding, contracting, or dissolving in response to changes in the energy state of the cell. Autophagy is a universal cellular function that facilitates the orderly breakdown and recycling of damaged or harmful components, hence preserving intracellular nutrition and homeostasis.

Lipophagy is defined as the autophagic degradation of lipid droplets. It is a progress of selective autophagy that can transport intracellular lipids to lysosomes for degradation and recycling. It also helps to regulate intracellular lipid storage, levels of free fatty acid, and energy balance. Then the lipid metabolites are continuously circulated and redistributed to maintain the body's energy balance. Singh et al.'s groundbreaking 2009 study in hepatocytes revealed that autophagy contributes to the degradation of LDs and proposed the concept of “lipophagy.” This progress is complex and directly or indirectly affected by genes, enzymes, transcriptional regulators, or other factors. Lipophagy is essential for the storage and utilization of energy, which explains its significance in various organisms, cell types, metabolic states, and diseases. With the changes of human living environment, social environment and lifestyle, research has revealed that lipophagy was not only closely associated with hepatopathy but also played a significant role in metabolic disorders such as metabolic syndrome, atherosclerosis, and cancer. These studies have stimulated researcher's interest in the role of lipophagy in the pathogenesis and treatment of diseases. Targeting lipophagy therapy will be a novel therapeutic strategy and important regulator for some related diseases.

Despite the fact that lipophagy research is still in its infancy, recent advances have substantially expanded our knowledge of this crucial lipid metabolic process. Over the last ten years, the overall number of articles on lipophagy has increased dramatically. Using conventional approaches to capture the development of lipophagy is becoming more and more challenging. Therefore, this article with the help of bibliometric tools transforms the data of lipophagy articles in the past 10 years into visualize images, visualizing the network between lipophagy knowledge groups, exploring the evolution and cutting-edge areas of lipophagy, and finding the latest trends in lipophagy research.

## 2 Method

### 2.1 Data sources and statements

**Fig. 1:**
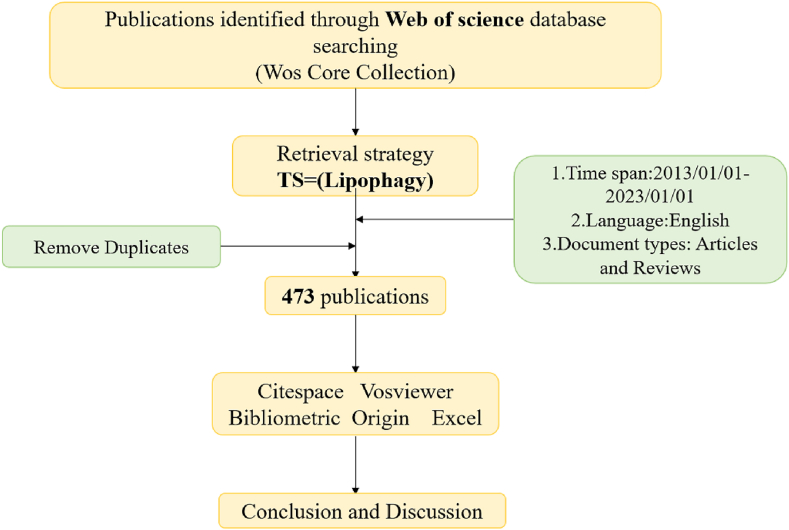
Flow chart.

The Web of Science database was used for the search. The time span was set between January 1, 2013, and January 1, 2023. The search topic was “lipophagy”, and the article types were “article” and “review”. Select “Plain text” as the file format The search query string is described as follows: TS=Lipophagy (Fig. 1).

### 2.2 Data analysis

To guarantee data accuracy and reproducibility, two researchers downloaded and examined the data. Utilize Origin 2021 and Microsoft Excel 2019 to analyze the target files, then export line charts and tables showing the authors, institutions, countries/regions, references, and keywords that have the most productivity or citations.

### 2.3 Bibliometric analysis and visualization software

Citespace (https://citespace.podia.com/download,R6.1.6) is a free Java-based application designed to analyze and visualize trends and patterns in scientific literature, presenting the structure and distribution of scientific knowledge. Description of the Citespace parameter configuration used this time: (1) Time zone selection: Time slicing is from 2013 to 01-01 to 2023-0101, and years per slicing is 1. (2) Threshold: The default value of the g-index parameter is k = 25, and TopN is 50. (3) Select the Cosine method for the node correlation strength Links parameter. (4) Clustering: LSI algorithm and LLR algorithm to extract research terms according to needs.

Furthermore, Citespace provides two indicators, the module value (Q value) and the average silhouette value (S value) based on the network results and the clarity of clustering, which are used as the basis for us to judge the map drawing effect. Q > 0.3 means that the divided structure is significant, when the S value is 0.7, the clustering is highly efficient and convincing. If the S value is above 0.5, the clustering is generally considered reasonable. Automatic clustering and automatically extracted cluster tag g words greatly help us understand the content of the network's content. When we understand the network structure and content, it is essential to find special points and connecting lines. Finding special points can be based on betweenness centrality, burst, centrality of mediation, and sigma value (Σ). In this study, Citespace was used to find important literature nodes, correlations, and to find turning points, key points, and research hotspots in lipophagy research.

VOSviewer is a bibliometric analysis software used to draw knowledge maps, it can be used for analyzing co-authorship, co-occurrence, and co-citation, and visualizing the results. VOSviewer provides three visualizations, including network visualization, overlay visualization, and density visualization. (1) In network visualization, the more important the items, the larger the label and circle. The number and thickness of the connections between items represent whether the connections between the items are close. The greater the number and thicker the connections, the closer the connections between the items. (2) Overlay visualization colors the items, and the default color ranges from blue to green to yellow. (3) In density visualization, the color is same as overlay visualization, the color is from blue to green to yellow, and the density is from small to large. In this research, VOSviewer was used to visualize the authors, and institutions, to analyze the research results of lipophagy through three visualization maps.

## 3 Results

### 3.1 Global publishing trends

**Fig. 2:**
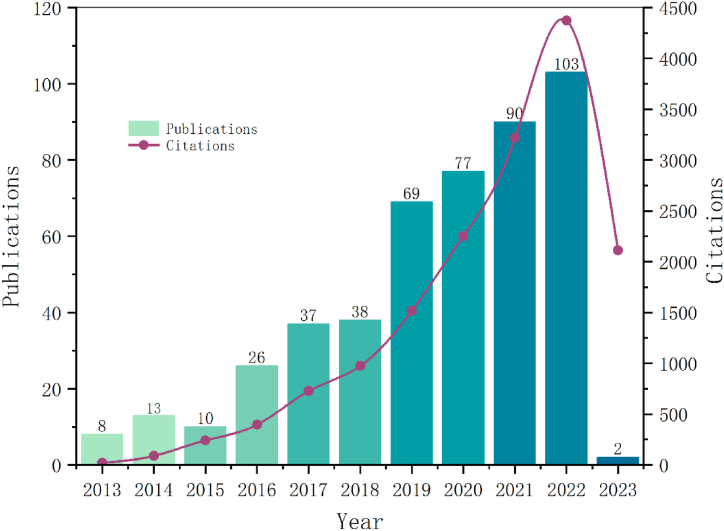
Global publishing trends.

A total of 534 papers were retrieved, 61 papers were eliminated due to conference abstracts, editorial materials, book, 473 valid papers were finally included. As Fig. 2 shows, the number of studies on lipophagy that have been published over the past ten years has been steadily rising. The years 2015–2016 and 2018–2019 saw a spike in the quantity of papers published, this was attributed to the ongoing discovery of lipophagy research.

### 3.2 Analysis of distribution and cooperation in major countries/regions

**Fig. 3:**
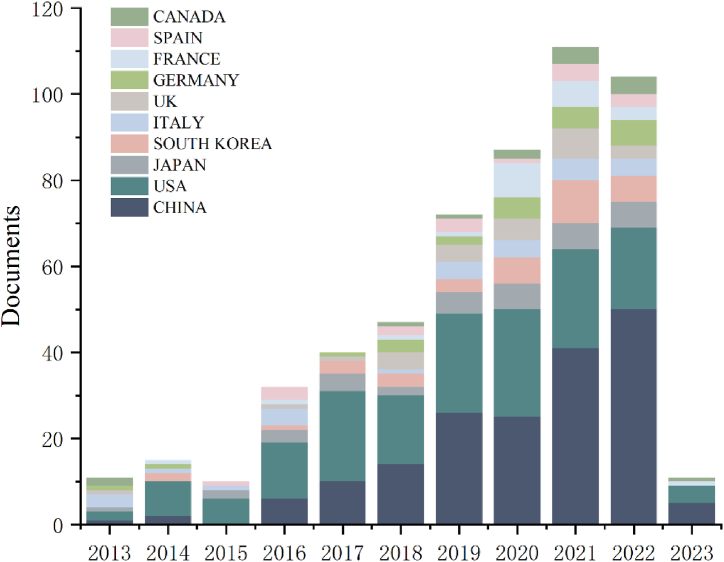
Number of publications and trend chart of lipophagy research by country.

A total of 65 countries/regions provided papers for lipophagy research. China (n = 172,36.36 %) has the highest number of publications, followed by the United States (n = 159,33.62 %), Japan (n = 35,7.40 %), South Korea (n = 33,6.98 %), Italy (n = 26,5.50 %), the United Kingdom (n = 24,5.07 %), Germany (n = 23,4.86 %), France (n = 21,4.44 %), Spain (n = 16,3.38 %), Canada (n = 14,2.96 %). The United States (n = 8730) has the highest total number of citations, followed by China (n = 3959), France (n = 1369), Italy (n = 1176), Sweden (n = 1036), the United Kingdom (n = 951), South Korea (n = 817), Japan (n = 655), Germany (n = 630), and Australia (n = 463) (Table 1) (Fig. 3).

**Table 1:** Top 10 countries/regions for publications and citations.

Rank	Countries/Regions	Publications	%（of 473）	Rank	Countries/Regions	Citations
1	CHINA	172	36.36 %	1	USA	8730
2	USA	159	33.62 %	2	CHINA	3959
3	JAPAN	35	7.40 %	3	FRANCE	1369
4	SOUTH KOREA	33	6.98 %	4	ITALY	1176
5	ITALY	26	5.50 %	5	SWEDEN	1036
6	ENGLAND	24	5.07 %	6	ENGLAND	951
7	GERMANY	23	4.86 %	7	SOUTH KOREA	817
8	FRANCE	21	4.44 %	8	JAPAN	655
9	SPAIN	16	3.38 %	9	GERMANY	630
10	CANADA	14	2.96 %	10	AUSTRIA	463

**Fig. 4:**
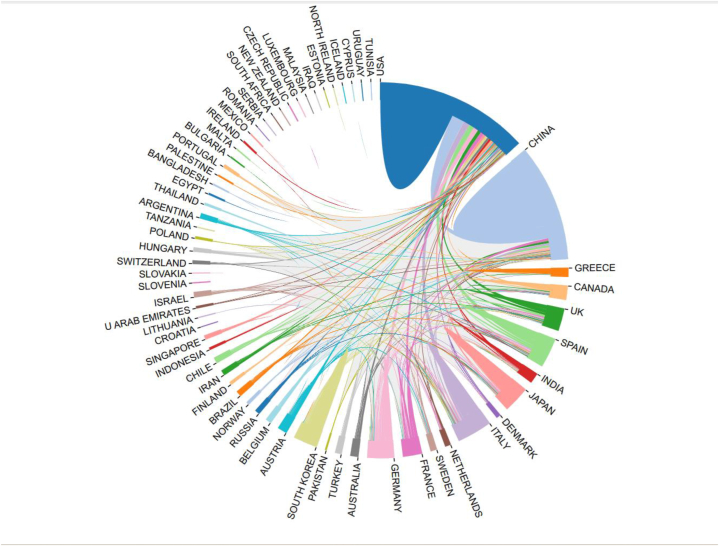
The bibliometric website created a graph of the relationship between lipophagy research publications in various countries.

Fig. 4 shows the United States has the most connections with other countries, indicating the most cooperation, followed by China. The United States and China cooperate more frequently with other countries, and cooperation with other countries is relatively weak. The United States and China, which account for 69.98 % of all publications, are two countries/regions that are tightly connected and that first developed a cooperation network (Fig. 4).

### 3.3 Analysis of institutions

There are many institutions involved in lipophagy research, among which Zhejiang University (n = 45) published the most papers, followed by Mayo Clinic (n = 42), Albert Einstein College of Medicine (n = 33), and University of Nebraska Medical Center (n = 30). The institution with the most citations is Albert Einstein College of Medicine (n = 745), followed by Mayo Clinic (n = 613), University of Minnesota (n = 418), and University of Nebraska Medical Center (n = 354). The papers with the highest average number of citations are Yeshiva University Albert Einstein College of Medicine (Frep = 64.00) and Albert Einstein College of Medicine (Frep = 22.58). (Table 2).

**Table 2:** Top 10 institutions for publications and citations.

Organizations	Article Counts	Total number of citations	Citations Per Paper	Total Number of First Authors	Number of citations of the first author	The average citation of the first author
Albert Einstein Coll Med	33	745	22.58	5	172	34.40
Mayo Clin	42	613	14.60	10	278	27.80
Univ Minnesota	23	418	18.17	5	90	18.00
Univ Nebraska Med Ctr	30	354	11.80	2	3	1.50
Zhejiang Univ	45	196	4.36	4	1	0.25
Yeshiva Univ Albert Einstein Coll Med	3	192	64.00	1	78	78.00
Shanghai Jiao Tong Univ	23	152	6.61	3	28	9.33
Baylor Coll Med	14	151	10.79	2	66	33.00
Univ Penn	23	149	6.48	1	1	1.00
Washington Univ	24	147	6.13	1	12	12.00

**Fig. 5:**
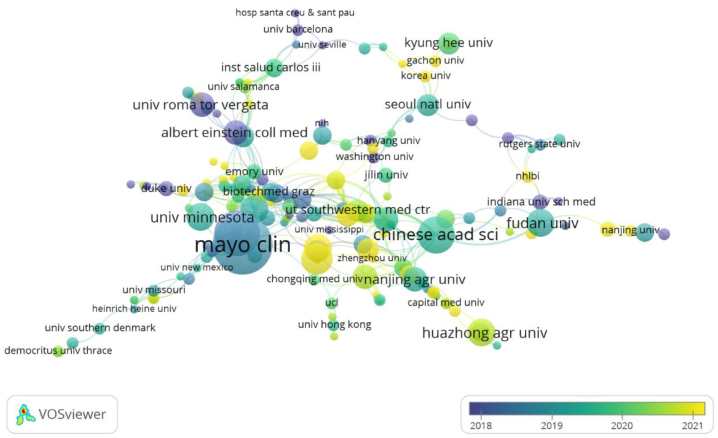
Institutions overlay visualization.

Based on the number of publications and citations, in Fig. 5, it is evident that the Mayo Clinic, Albert Einstein College of Medicine, and University of Minnesota are the three institutions that conduct the most lipophagy research and have the greatest impact on their findings (Fig. 5).

### 3.4 Analysis of keywords, bursts

**Fig. 6:**
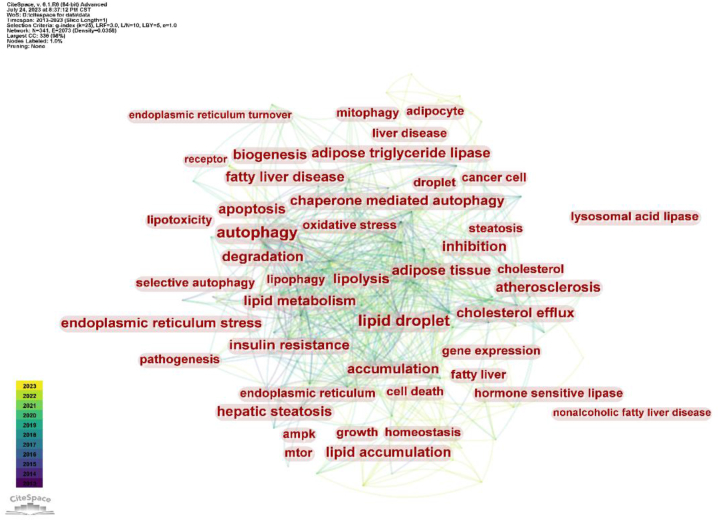
Visualization of important node keywords.

**Fig. 7:**
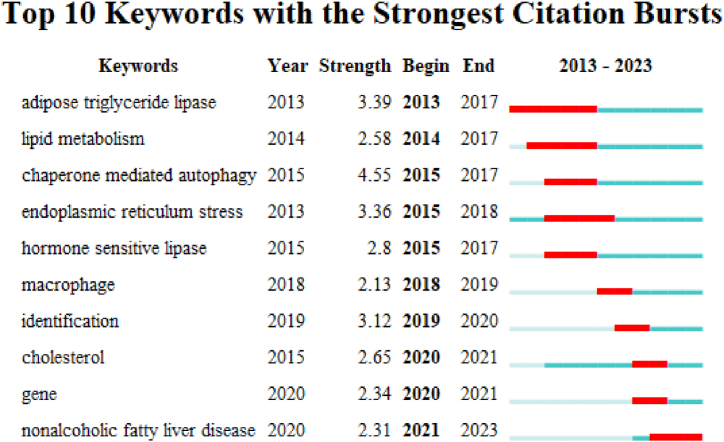
Top10 keywords with the strongest citation bursts.

Based on frequency, betweenness centrality, and Σ value, the hot keywords are autophagy, lipid droplet, lipid metabolism, degradation, metabolism, insulin resistance, lipolysis, chaperone-mediated autophagy, apoptosis, and endoplasmic reticulum stress (Table 3). Cooperating with Citespace top10 keywords with the strongest citation bursts in Fig. 7, it can be found that since lipophagy was studied in the field of lipid catabolism, research hotpots have focused on lipophagy-related disease and the mechanism of lipophagy (Fig. 6, Fig. 7).

**Table 3:** Top 10 based on keyword frequency, centrality, and Σ value.

Rank	Keywords	Frequency	Centrality	Σ
1	autophagy	165	0.12	1
2	lipid droplet	131	0.13	1
3	lipid metabolism	74	0.11	1.32
4	degradation	48	0.13	1
5	metabolism	74	0.07	1
6	insulin resistance	44	0.06	1
7	lipolysis	51	0.03	1
8	chaperone mediated autophagy	33	0.06	1.32
9	apoptosis	50	0.08	1
10	endoplasmic reticulum stress	45	0.07	1.27

### 3.5 Co-cited documents

**Fig. 8:**
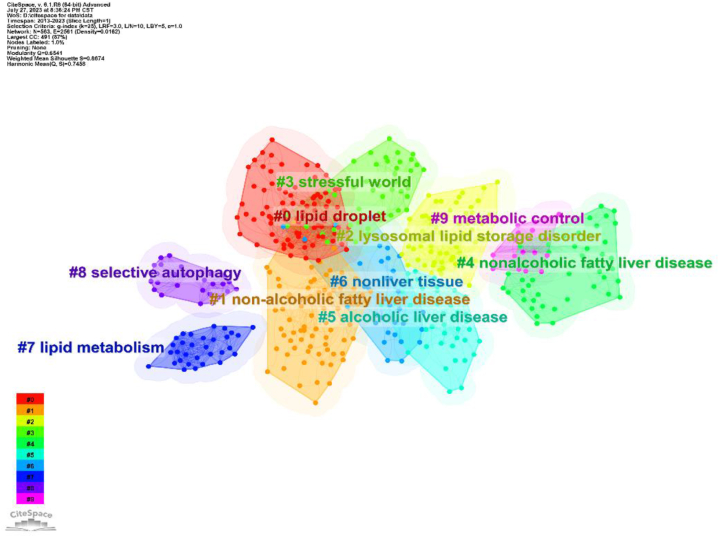
Cluster map analysis of co-cited papers.

The cluster figure of cited documents shows that the top10 cluster labels include: 0# lipid droplet, 1#non-alcoholic fatty liver disease, 2#lysosomal lipid storage disorder, 3#stressful world, 4#nonalcoholic fatty liver disease, 5# alcoholic liver disease, 6#nonliver tissue, 7#lipid metabolism, 8#selective autophagy, 9#metabolic control. Combining the top5 co-cited papers and cluster figure, it can be seen that the mainstream research is mainly in lipid metabolism and liver diseases (Table 4) (Fig. 8).

**Table 4:** Top 5 cited papers.

Rank	Name	Author（year）	Times Cited in All Databases	Source	IF/JCR（2023）
1	**Autophagy at the crossroads of catabolism and anabolism**	Kaur,J&Debnath,J（2015）	813	*Nature reviews. Molecular cell biology*	112.7（Q1）
2	**Degradation of lipid droplet-associated proteins by chaperone-mediated autophagy facilitates lipolysis**	Kaushik,S&Cuervo,AM（2015）	509	*Nature cell biology*	21.3（Q1）
3	**Autophagy-Dependent Ferroptosis: Machinery and Regulation**	Liu, J, Tang, DL（2020）	375	*Cell chemical biology*	8.6（Q1）
4	**Cytosolic lipolysis and lipophagy: two sides of the same coin**	Zechner,R (（2017）	339	*Nature reviews. Molecular cell biology*	112.7（Q1）
5	**Multiple Roles of the Small GTPase Rab7**	Guerra,F&Bucci,C（2016）	288	*Cells*	6.0（Q2）

## 4 Discussion

### 4.1 Published papers

Since Singh et al. proposed “lipophagy” in 2009 [1], researchers have progressively started delving into the significance of autophagy in the breakdown and use of lipids.

As a result, there have been progressively more papers in the subject of lipophagy study.

Fig. 2 shows that published papers increased most throughout the years 2015–2016 and 2018–2019, respectively (Fig. 2). The reason behind this can be known from the cited publications (Table 4). In 2015, a widely referenced work, Autophagy at the crossroads of catabolism and anabolism, was published by Kaur J and Debnath J. The article highlighted how advancements like lipophagy, gulcophagy, and ferritinophagy allow cells to salvage critical metabolites to maintain and promote core anabolic functions, thereby elucidating the established and emerging role of autophagy as a process in promoting biosynthetic capacity and promoting metabolic and nutritional homeostasis [2]. Regarding the increase in the number of published articles from 2018 to 2019, by interpreting the appearance time of burst words and counting the titles of articles in 2017 and 2018, Maan, M. et al.'s paper, “Lipid metabolism and lipophagy in cancer,” was the most highly cited of that year [3]. The area of abnormal tumor lipid metabolism is relatively recent. As an alternate route for the destruction of lipid droplets, lipophagy plays a crucial role in cancer and metastasis due to its participation in lipid turnover. Since then, lipophagy has been linked to cancer and has garnered significant attention, following 2019, the number of publications on lipophagy and cancer has increased even further.

### 4.2 Countries, regions and institution

The United States is the first country put forward to research lipophagy, has made research contributions to lipophagy types, mechanisms, and diseases, and has intimate cooperation with other countries (Table 1), including the Albert Einstein College of Medicine, Mayo Clinic, University of Minnesota and other institutions are major research institutions (Table 2). As a result of the aged population trend and high incidence of metabolic diseases in China, Japan, and South Korea, there are also an increasing number of research institutions targeting lipophagy, such as Zhejiang University and Fudan University in China, Seoul National University and Korea University in South Korea, however, there is relatively little cooperation between countries, and research on lipophagy needs further communication and exchange. At the same time, some countries, such as Japan and South Korea, rank at the top in terms of the number of publications, but lack citations. This may be due to the fact that the content of the research on lipophagy in the above two countries is relatively single. For instance, research on the application of lipophagy to a single medication ingredient in the treatment of a particular disease is more common in Japan, and the research content is scattered. Second, the lipophagy related illnesses under investigation, like breast and ovarian cancer, are rather uncommon. Lastly, Fig. 4 illustrates how less cooperative the two nations are with other countries than China and the United States. Consequently, even though the aforementioned nations lead the world in publications, there may be some variation in the quantity of citations (Fig. 4, Fig. 5).

### 4.3 Research frontiers and hot spots

#### 4.3.1 Types of lipophagy

Autophagy as a cellular mechanism can orderly digest cellular components [4]. The are three types of autophagy: macroautophagy, chaperone-mediated autophagy, and microautophagy. Macroautophagy is the process of wrapping and degrading large intracellular structures through the formation and fusion of vesicles. Macroautophagy begins with membrane expansion and protein aggregation. It first forms a double-membrane vesicle (autophagosome), then the organelles or proteins to be degraded are wrapped in the cytoplasm, and the vesicles fuse with lysosomes and are broken down by degrative enzymes [5]. Chaperone-mediated autophagy (CMA) is a highly selective autophagy process that targets specific protein molecules [6]. In the cytoplasm, a specific chaperone protein will recognize and bind to the target protein that needs to be degraded. The target protein carries a KFERQ signal to ensure that the target protein is recognized by the chaperone protein. The complex formed by the combination of the target protein and the chaperone protein binds to the lysosome-associated membrane protein 2A (LAMP-2A), and is guided into the interior of the lysosome, where it is eventually degraded [7]. Microautophagy directly wraps up a small part of the cytoplasm through vesicles or membrane structures and send it to lysosomes for degradation. Compared with the other two forms of autophagy, microautophagy is a relatively less studied form of autophagy, but it still plays important degradative and regulatory functions within cells [8]. The goal of autophagy, in whatever form, is to remove and degrade aging or damaged organelles, proteins, and other cellular components within cells; it also releases raw materials and energy for subsequent cell use via lysosome degrades and preserves cellular homeostasis.

Since 2009, Singh et al. proposed the category of lipophagy as macroautophagy and found that during nutrient deprivation, inhibition of autophagy in hepatocytes and mouse livers increases triglyceride (TG) and LDs in vitro and in vivo, which means the loss of autophagy reduces the decomposition of TG. Studies using electron microscopy have demonstrated that autophagosomes phagocytose LDs or a small portion of large LDs for degradation to form autophagosomes, a process that is currently referred to as macrolipophagy [1]. This study identified the key function of macroautophagy in lipid metabolism and has since opened the door to the study of lipophagy. Subsequently, improper macrolipophagy in parenchymal and non-parenchymal liver cells is linked to the involvement of macrolipophagy in metabolic illnesses such fatty liver diseases and nonalcoholic fatty liver disease (NASH) [9].

For the chaperone-mediated molecular autophagy type of lipophagy, Fig. 7 illustrates that it first appeared as a research hotspot in 2015. In 2015, Kaushik, S. and Cuervo, AM, both from the Albert Einstein College of Medicine, published a highly cited paper on lipophagy in which they proposed the role of CMA in lipidbiology and maintaining lipid balance [10]. According to this article, lipolysis is encouraged by CMA degradation of proteins linked to lipid droplets. Studies conducted in vivo have verified that the degradation of CMA using the LD-associated proteins perilipin 2 (PLIN2) and perilipin 3 (PLIN3) as substrates is accelerated under starvation conditions. At the same time, blocking CMA or expressing CMA-resistant PLINs in cultured cells and mouse livers reduces lipid oxidation and the accumulation of LDs. As research deepens, the role of CMA in another disease of abnormal lipid metabolism-atherosclerosis, has also been discovered. To control cellular lipid and glucose metabolism, CMA can quickly destroy restriction enzymes and other proteins involved in metabolism [11]. With aging and chronic lipid overload, the activity of CMA declines, which represents a significant risk factor for the development of atherosclerosis.

Compared to macroautophagy and CMA, microautophagy has received less attention in the realm of lipophagy, nonetheless, Schulze et al. first discovered a mechanism similar to the autophagy mechanism of yeast cells, which involves the direct transfer of lipid droplets to lysosomes for degradation. Their findings, which were published in the PANS journal, unequivocally demonstrated how nutritional restriction alters the vacuole membrane to facilitate microautophagy. Microautophagy, as a key mechanism of deprivation, proves that microautophagy is a necessary mechanism in consuming LDs [12]. Joel M. Goodman then went over the significance of hepatic macrolipophagy and clarified that even though macrolipophagy in complete lipid droplets is comparatively hard to observe, aside from small lipid droplets, this does not negate the significance of macrolipophagy in hepatic lipid metabolism [13].

Lipid droplets are dynamic lipid storage organelles that can be degraded by autophagy to release neutral lipids. Lipophagy includes the formation of pre-autophagosome structures, autophagosome recognition and engulfment of LDs mediated by specific receptors, and fusion of autophagosomes with lysosomes for degradation. Regarding the regulator mechanism of lipolysis, in addition to the above three types that have been introduced, the mechanism of how to trigger lipophagy is still mostly unknown. Consequently, as shown in the keywords screened in Fig. 6, there has been study and attention focused on the particular receptor mechanism of lipophagy in recent years. From an enzymatic standpoint, for instance, lipophagy and the cytosolic lipases ATGL and HSL contribute to LDs mobilization, and cold induces autophagy in proopiomelanocortin (POMC) neurons and activates lipophagy in brown adipose tissue (BAT) and liver in mice, thus advancing lipolysis in peripheral tissues [14]. From a genetic perspective, ATG14 is a core component of the autophagy initiation complex class III phosphatidylinositol 3-kinase complex 1 (PI3KC3–C1), which can act as a fat autophagy receptor to promote lipid droplet degradation. The negative regulatory factor Syntaxin18 (STX18) binds to ATG14, destroying the interaction between ATG14-ATG8 family members and the formation of the PI3KC3–C1 complex, thereby preventing lipophagy [15]. As a recognized endoplasmic reticulum lipid transfer protein, oxysterol-binding protein (OBP)-related protein 8 (ORP8) localizes to lipid droplets and controls the encapsulation of LDs by autophagosome membranes, thereby targeting and regulating the process of lipophagy [16]. Subsequent investigations disclosed that the master regulator of autophagy, mammalian target of rapamycin complex (mTORC) 1 and the LD coating protein perilipin (Plin) 3 in was found to function as a docking protein and participate in autophagosome formation to activate lipophagy [17]. During this research process, some controversial receptors were also discovered and studied. For instance, the spartin protein was discovered by Jeeyun Chung's research group to be a lipophagy receptor [18]. Still unanswered are topics such why spartin exclusively targets a subset of lipid droplets, how to cause lipid droplet degradation caused by spartin, and whether or not spartin mediates lipophagy in different tissues and cell types [19]. Thus, future study on the mechanism triggering lipophagy and lipophagy receptors is also warranted.

#### 4.3.2 Hotspot diseases of lipophagy

##### 4.3.2.1 Fatty liver

Hepatic steatosis is a pathological condition in which vacuoles of triglyceride (TG) fat excessively accumulate in the liver due to abnormal lipid metabolism. Hepatic autophagy regulates lipid metabolism, and regulating fat has become a new therapeutic strategy for fatty liver disease [20].There are two types of fatty liver: alcoholic fatty liver and nonalcoholic fatty liver. In both cases, impaired lipophagy will lead to the progression of the disease. Under normal circumstances, lipophagy in liver cell actively organizes lipid droplets aggregation. Long-term drinking can destroy this fat burning function by slowing down lipophagy and accelerating lipogenesis, leading to the formation of alcoholic fatty liver [21,22].Meanwhile, the lack of lipophagy in nonalcoholic fatty liver disease leads to the occurrence of excessive triacylglycerol (TAG) induced lipotoxicity, resulting in the progression of fatty liver [23].

Based on the important role of lipophagy in fatty liver, the treatment of fatty liver with lipophagy has become a hot topic in recent years [23].The key negative regulator of lipophagy, mTORC1, has recently become a targeted regulator of nonalcoholic fatty liver disease [24].The antidiabetic drug metformin can mediate tristetraprolin (TTP) to inhibit mTORC1, promote hepatocyte lipophagy, and alleviate hepatic steatosis [25]. Annexin A2 (ANXA2) plays a crucial role in linking inflammation to liver metabolic disorders and damage and has been shown to block AMPK/mTOR pathway-mediated lipophagy, promoting lipid accumulation and liver damage, making ANXA2 a pathological predictor and promising therapeutic target for nonalcoholic fatty liver disease [26]. In addition to drug therapy, exercise is a non-pharmacological treatment for fatty liver disease by modulating lipophagy [27].Exercise and dietary intervention can enhance lipophagy and reduce the formation of lipid droplets by activating AMPK/ULK1 and inhibiting Akt/mTOR/ULK1 pathway respectively, this study also provides evidence to support that muscle exercise is beneficial to other metabolic organs such as the liver, thereby improving nonalcoholic fatty liver disease and the progression of aging [28,29]. According to clinical research, zinc helps regulate the oxidant/antioxidant system and enhances lipid metabolism in a variety of illnesses. Studies both in vivo and in vitro have shown that zinc inhibits hepatic lipid accumulation and has a specific impact on lipophagy. To ascertain the beneficial effects of zinc supplementation on serum zinc and liver enzyme levels in overweight/obese patients with NAFLD, Fathi, M. et al. used a randomized clinical trial to study the effects of zinc supplementation on the clinical manifestations and anthropometric parameters of overweight/obese patients with NAFLD following a calorie-restricted diet [30].

##### 4.3.2.2 Atherosclerosis

Atherosclerosis is an inflammatory disease caused by the accumulation of cholesterol-rich plaques in artery walls. The progression of atherosclerosis can be slowed by removing cholesterol from the plaques and/or reducing inflammation [31]. In the progression of atherosclerosis, lipophagy plays a regulatory role in vascular endothelial cell damage, vascular smooth muscle cell phenotype shift, and macrophage lipid accumulation. Among them, the research hotspot of the relationship between lipophagy and atherosclerosis lies in macrophages, which is reflected in the bursts keywords (Fig. 7). For example, macrophages can selectively activate lipophagy against atherogenic lipoproteins, thereby reducing the occurrence of atherosclerosis. At the same time, the acid-sensing ion channel 1 (ASIC1) of macrophages inhibits lipophagy in interaction with receptor-interacting protein 1 (RIP1), thereby promoting atherosclerosis formation. Targeting ASIC1 therapy represents a promising therapeutic approach for atherosclerosis [32]. In addition, in the study of PCSK9 preparations that are now widely used in clinical practice, it was found that PCSK9 mediates lipophagy and promotes lipid degradation, making it an important mechanism for the treatment of atherosclerosis [33]. Another widely used lipid-lowering drug-atorvastatin, promotes lipophagy, cholesterol efflux, reduces lipid accumulation, and inhibits the formation of foam cells, the main pathological component of atherosclerosis, by upregulating AMPK phosphorylation and downregulating mammalian target of rapamycin phosphorylation [34].

##### 4.3.2.3 Cancer

From the respective of lipophagy and cancer, metabolism-related cancer research has received increasing attention in the past decade. In particular, the fatty acids produced by lipophagy are important energy source for β-oxidation. Cancer cells can use this energy to continue to grow and proliferate, which has led to more and more attention to the role of lipophagy in cancer [3,[35], [36], [37]].Among the liver diseases most closely related to lipophagy, when the endoplasmic reticulum resident protein (Nogo-B) is overexpressed in NAFLD-related hepatocellular carcinoma, it can activate lipophagy, degrade TG, and increase the content of free fatty acids, inducing carcinogenicity [38]. Overactivated transcription factor CCAAT enhancer binding protein α (C/EBPα) reduces liver tumorigenesis by activating lipophagy [39]. Newer studies have also found that caner-associated fibroblasts can promote hepatocellular carcinoma recurrence and metastasis through CD36-mediated fatty-acid metabolism reprogramming [40].

In recent years, the study of lipophagy in different cancer fields has gradually become a hot topic, including but not limited to brain tumor cells [41], renal cell carcinoma [42], and reproductive-related cancers. Duc-Vinh Pham and Pil-Hoon Park found that adiponectin stimulated lipophagy-mediated lipolysis and fatty acid oxidation, demonstrating that fatty acid metabolic reprogramming triggers breast cancer cell death [43]. The lipophagy-ICAM-1 pathway induced under tumor-like stress conditions contributes to the progression of ovarian clear cell carcinoma, making the lipophagy pathway a potential therapeutic target for this aggressive cancer type [44]. Periprostatic adipose tissue (PPAT) plays an important role in the progression of prostate cancer, and lipophagy is consider to be a key process in the progression of prostate cancer [45].

##### 4.3.2.4 Neurodegenerative diseases

Research has indicated that lipophagy contributes significantly to neurodegenerative illnesses. Abnormal lipid accumulation and metabolism can serve as indicators for disorders related to the nervous system. By lowering activity-dependent neurodegeneration in neural tissue, lipophagy ameliorates neurodegenerative illnesses [46]. Disorders related to lipid metabolism pose a danger for neurological conditions including Alzheimer's disease (AD) [47]. Cutler et al. discovered that the development of clinical AD can be attributed to the neurodegenerative cascade brought on by the accumulation of cholesterol and ceramide species. Wei Hung Jung et al. discovered that lipophagy can prevent the body's accumulation of dihydroceramide from causing activity-dependent neurodegeneration [48]. According to a recent study by Hao Huang et al. RTN3, a tubular endoplasmic reticulum protein, may interact with heat shock cognate protein 70 (HSC70) to reduce its function in chaperone-mediated lipophagy, which in turn may promote the enrichment of neutral lipids near plaques and mediate lipid accumulation in AD neuropathology [49]. Natural flavonoid small molecule kaempferol (Ka) prevents dopaminergic neuron degeneration in Parkinson's disease by promoting lipophagy, which consequently inhibits lipid peroxidation-mediated mitochondrial damage. This suggests a potential new therapeutic approach for Parkinson's disease and related neurodegenerative diseases [50]. Thus, lipophagy is going to be an increasingly important field in neurodegenerative research in the future.

##### 4.3.2.5 Hyperlipidemia

One separate risk factor for atherosclerosis is hyperlipidemia [51]. It is thought to be a condition of lipid metabolism linked to high levels of cholesterol and/or plasma triglycerides, and it plays a significant role in the development of cerebrovascular and cardiovascular diseases. Research has indicated that the degradation of triacylglycerols, cholesterol, and other lipids is impacted by defective lipophagy, which can result in hyperlipidemia. The ability of mice to withstand hepatic lipidosis and hypercholesterolemia brought on by high-fat/high-cholesterol diet can be improved by inhibiting mTORC1 activity in the liver [52]. Hyperlipidemia has been linked to altered bone tissue homeostasis, according to more recent research. Lipophagy activation partially restores osteoblast function under high physiological lipid circumstances, offering fresh hope for treating lipid metabolic disorders-related bone failure [53].

## 5 Conclusion

Autophagy is a cellular mechanism with a two-sided nature. Under starvation conditions, autophagy non-selectively promotes the degradation of cytoplasmic components, while under nutrient-rich conditions, autophagy selectively eliminates specific cytoplasmic cargoes [54]. With this property, With this ability, lipophagy contributes to the removal and degradation of lipid droplets and other organelles, preserving the body's normal ability to produce energy and maintain homeostasis within the intracellular environment. The idea that “a research field can be conceptualized as a time mapping Φ(t) from the research frontier Ψ(t) to the knowledge base Ω(t)” allows bibliometrics to help us sort through the vast amount of literature on lipophagy and identify trends, developments, key nodes, and research frontiers in the field.

Remarkably, more and more countries and institutions are delving deeply into lipophagy research. Through the three forms of macroautophagy, microautophagy, and chaperone-mediated molecular autophagy, this intricate cellular mechanism known as lipophagy has demonstrated its potential therapeutic practicality in the treatment of fatty liver, atherosclerosis, and cancer. While cancer and neurodegenerative diseases are currently in the stage of in vivo and in vitro animal testing, medications related to fatty liver, atherosclerosis, and hyperlipidemia have been clinically studied and applied in clinical practice, offering treatment options for the front line of clinical practice. However, the development of medications for equivalent therapeutic targets has risen to the forefront of research due to the significant finding of lipophagy in cancer and neurological diseases.

At the crossroads of lipophagy research, where is the future of lipophagy research? Important references and research horizons are supplied through the presentation and interpretation of the lipophagy map, encouraging additional study and advancement in the field of lipophagy and offering therapeutic suggestions for medical conditions.

## 6 Deficiencies

Initially, we only included data from the WoSSC database in the dataset, which would have excluded any pertinent studies from other sizable databases. Second, by excluding non-English literature, we disregarded the contributions that some non-English-speaking regions have made to this discipline. Third, the influence of newly released articles on the field can be understated because of the database's constant updating. Fourth, a literature with a lower frequency of appearance cannot be given a high value in bibliometrics due to the vast quantity of literature in the field.

## Data availability statement

This data is not applicable.

## Ethics statement

This research did not involve any human subjects, and the Web of Science database (WOS, https://www.webofscience.com/wos/) was used to perform bibliometric data analysis on the retrieve. This study was exempt from institutional ethics review board approval.

## Fund

This work was supported by the 10.13039/501100001809National Natural Science Foundation of China (No. 82374428); Scientific and technological innovation project of 10.13039/501100005892China Academy of Chinese Medical Sciences, No. CI2021A03323.

## CRediT authorship contribution statement

**Lu Zhao:** Writing – original draft, Visualization, Formal analysis, Data curation. **Mengmeng Pang:** Writing – original draft, Visualization, Software, Data curation. **Zhenyue Fu:** Writing – original draft, Software, Data curation. **Huaqin Wu:** Writing – review & editing, Funding acquisition. **Qingqiao Song:** Writing – review & editing, Funding acquisition.

## Declaration of competing interest

The authors declare that they have no known competing financial interests or personal relationships that could have appeared to influence the work reported in this paper.
